# Growth of fungi and yeasts in food production waste streams: a feasibility study

**DOI:** 10.1186/s12866-023-03083-6

**Published:** 2023-11-06

**Authors:** D. Bansfield, K. Spilling, A. Mikola, J. Piiparinen

**Affiliations:** 1 Marine and Freshwater Solutions, Finnish Environment Institute, Agnes Sjöbergin katu 2, Helsinki, 00790 Finland; 2 Department of Built Environment, Aalto University, Tietotie 1E, Espoo, 00076 Finland; 3 Centre for Coastal Research, University of Agder, Universitetsveien 25, Kristiansand, 4630 Norway

**Keywords:** Biomass, Water reuse, Circular economy, Fungi, Side streams, Sustainability, Yeasts

## Abstract

Food production produces nutrient-rich waste streams which, depending on local legislation, are either sent to wastewater treatment plants or discharged into the environment. In addition to causing environmental harm in the second instance, valuable nutrients are lost. A more circular approach would be to reuse these waste streams. Fungi and yeasts are ideal candidates as they require lots of organic carbon (which is especially high in food production waste streams) for growth, with the potential for producing value-added biomass. Here, we tested the metabolic activity and possible growth of seven fungi and three yeasts in five different food production waste streams. Initial tests were done to find the most promising waste streams for growth and these were chosen for further study. All species were then cultured in these waste streams and oxygen uptake was measured to gauge metabolic activity which we used as a proxy for growth rate. Pelletization’s effect on metabolic rates was tested on the most pellet-forming species, by adding agar to inhibit pellet formation. The most promising waste stream for yeast/fungal growth was cheese whey (Whey). Pellet inhibition (i.e., filamentous growth) resulted in increased metabolic activity of cells in the confectionary bakery waste stream with agar but decreased metabolic activity in Whey with agar. The best-growing species, *Geotrichum candidum*, has potential commercial value as a producer of enzymes, biochemicals and lipids and could provide added value while improving the circularity of water and nutrients in food production.

**Supplementary Information:**

The online version contains supplementary material available at 10.1186/s12866-023-03083-6.

## Introduction

The world’s population hit a milestone of 8 billion in 2022 which will result in increased consumption of valuable raw materials such as water and nutrients. Due to climate change usable water has been in short supply in many regions and is predicted to decrease further in the future [1]. This is problematic as water is not only needed to sustain human life but is involved in all human activities and the production of goods [2]. Food production consumes enormous quantities of water (132–12 6505 m^3^ ton^− 1^ depending on the product) [3], concomitantly resulting in large quantities of wastewater [2, 4]. Later steps in food production, food processing, is particularly water-intensive [2], producing wastewaters high in carbohydrates that generate 45% of all industrial organic pollution [5]. Food production and processing waste streams (FPWS) may also be high in inorganic nutrients and fats, depending on the food product.

Globally, the nutrients in FPWS are poorly exploited as they are either discharged directly into the environment, or if treated, 80% is converted into bacterial biomass forming sludge which creates treatment and disposal costs [6]. The high Chemical Oxygen Demand (COD), inorganic nutrients, and fat content of FPWS combined are problematic for wastewater treatment plants (WWTPs) as they cause processing problems [6, 7]. However, these “problematic” nutrient-rich wastes represent an opportunity for the recovery of valuable raw materials. For instance, inorganic nutrients such as nitrogen and phosphorus, the main constituents of fertilizer, are limited due to extensive phosphorus rock mining [8], and nitrogen’s high industrial demand [9], resulting in scarcity and rising prices [10]. More sustainable solutions for FPWS are needed to reduce the environmental footprint of food production and advance its circularity.

Some of the main strategies for increased circularity of water in food processing are, (1) the reuse of white (very low in organics) and grey (heavier loads of organics, suspended solids and nutrients) wastewater [4, 11], (2) the recovery of valuable compounds, e.g. polyphenols, pectin, fibres, proteins, antioxidants, starch [12], and (3) biological methods, especially for nutrient recycling into value-added biomass [4]. Reuse of grey wastewater and recovery of compounds involve energy-intensive steps such as membrane filtration, flotation, or crystallization [4], and may encounter fouling problems in the case of membrane filtration. Therefore, they are the most complicated and least sustainable options as they require material and energy inputs which themselves produce waste [4, 12]. A more environmentally sustainable method of resource recovery is the use of biological methods. This employs the growth of bacteria, algae, fungi, or yeast in FPWS for nutrient recycling via the production of value-added biomass [4, 13]. The biomass produced can be used as feedstock for biogas and biofuel production, converted into feed or fertilizer, or for the production of a range of commercially important biochemicals that can further add value to the process [4, 6, 14].

Fungi and yeast require large quantities of organic carbon as an energy source for growth and are therefore good candidates for the conversion of FPWS high in organic content into value-added biomass [6, 15]. Additionally, fungi produce a broad range of enzymes enabling them to break down and use a variety of substrates (e.g., sugars, recalcitrant organics, ammonia, and even lipids) for metabolism and biomass growth [15–17]. Bioconversion of FPWS into high-value fungal and yeast biomass could be used as a pre-treatment for FPWS preventing nutrient overloading of WWTPs and reducing treatment costs. Fungi and yeasts produce a wide range of lipids, proteins, biochemicals, vitamins, and supplements [6]. They also produce many industrially important enzymes and have a balanced amino acid profile making it potentially nutritious [6, 18]. Additionally, filamentous fungi can be used for various specific purposes like flocculating microalgae [19], providing a cheap alternative for microalgal harvesting, which is currently an expensive process [20]. Fungal cultivation in FPWS for various applications increases sustainability and develops the circular economy of food processing plants, furthering the EU’s SDG 12.

Research into the use of fungi and yeasts for bioconversion of FPWS into value-added biomass and by-products has focused mainly on yeasts. Only a few filamentous fungal species such as *Trichosporon*, *Aspergillus*, and *Rhizopus* spp., have been studied. In addition, the types of food waste streams investigated have been few, concentrating on wastewater from the processing of carbohydrate-rich staples or vegetable oils [6, 21]. In our study seven filamentous fungi and three yeasts were tested for their ability to grow in five food production waste streams from malting, recirculating aquaculture, confectionary/bakery, and dairy industries. All fungal species are non-pathogenic [22], and have the potential to produce valuable by-products such as lipids, proteins, biochemicals, and enzymes [6]. Our main aim was to determine the growth potential of these fungi and yeasts in the five FPWS, and therefore, the feasibility of reusing these WS for fungal and yeast biomass production.

## Materials and methods

### Species and waste streams

Ten fungal/yeast strains were used in this study: *Ganoderma lucidum* (NRRL 66208), *Geotrichum candidum* (NRRL Y-552), *Geotrichum fermentans* (NRL Y-1492), *Lipomyces starkeyi* (NRL Y-11557), *Paecilomyces variotii* (NRRL 1115), *Penicillium corylophilum* (NRRL 802), *Penicillium restrictum* (NRRL 3381), *Pleurotus ostreatus* (NRRL 3526), *Trametes versicolor* (NRRL 66313), provided by the USDA-ARS Culture Collection (NRRL), and *Lentinus tigrinus* (HAMBI/FBCC 645) procured from The University of Helsinki’s Microbial Domain Biological Resource Centre, HAMBI. Upon receipt, each strain was transferred to Potato Dextrose Agar (PDA) plates (CM0139 Oxoid) and incubated at 24 °C for one week, transferred to 4 °C and refreshed every 3–4 months. Liquid suspensions were made by boring a 1 cm ø piece of agar from the outer edge of the fungal mass, suspending it in 20 mL of Yeast Malt Broth (YMB) (3.0 g of Yeast extract (LP0021 Oxoid, Thermo Fisher), 3.0 g Malt extract (LP0039 Oxoid, Thermo Fisher), 5.0 g Peptone (07751 Fluka analytical) and 10.0 g Glucose (G7021 Sigma-Aldrich) in 1 L of sterile MQ) in 250 mL culture flasks and incubating at 24 °C for 2 weeks. Liquid suspensions were then split by inoculating 18 mL of YMB with 2 mL of 2-week-old fungal culture and incubated at 24 °C for one week. All maintenance and inoculate suspensions were cultivated under static conditions.

Five waste streams with potentially high organic loads were procured from various food and beverage production and processing industries around Finland. These were barley steeping water (BSW), broad bean steeping water (PSW), cheese whey (Whey), wastewater from a confectionary and baked goods plant (CWS), and recirculating aquaculture sludge (RAS). All the WS were frozen upon collection and stored at − 20 °C until use. Each waste stream was thawed and autoclaved at 121 °C for 30 min before use. No additional nutrient sources such as carbon or nitrogen were added.

### Growth trials

An initial screening test was done in each waste stream to determine their potential as fungal/yeast growth media. Growth trials were set up by centrifuging 2 mL of each fungal/yeast suspension at 4000 rpm for 4 min, decanting the growth media (YMB) to ensure no extra addition of nutrients, and then resuspending the fungal pellet in 20 mL of each WS in a 50 mL cell culture flask and incubating it at 24 °C for up to 2 weeks. A negative control without fungi/yeast was also set up for each WS. Visual observations (turbidity, appearance of filaments, number of spots or size, and fungal mat thickness) were recorded at 2 days, 1-week, and 2-week time points. WS which resulted in abundant and continuous biomass increases of the most cultures over the 2-weeks were chosen for further study and analysed for nutrient content.

### Oxygen uptake measurements

**Fig. 1:**
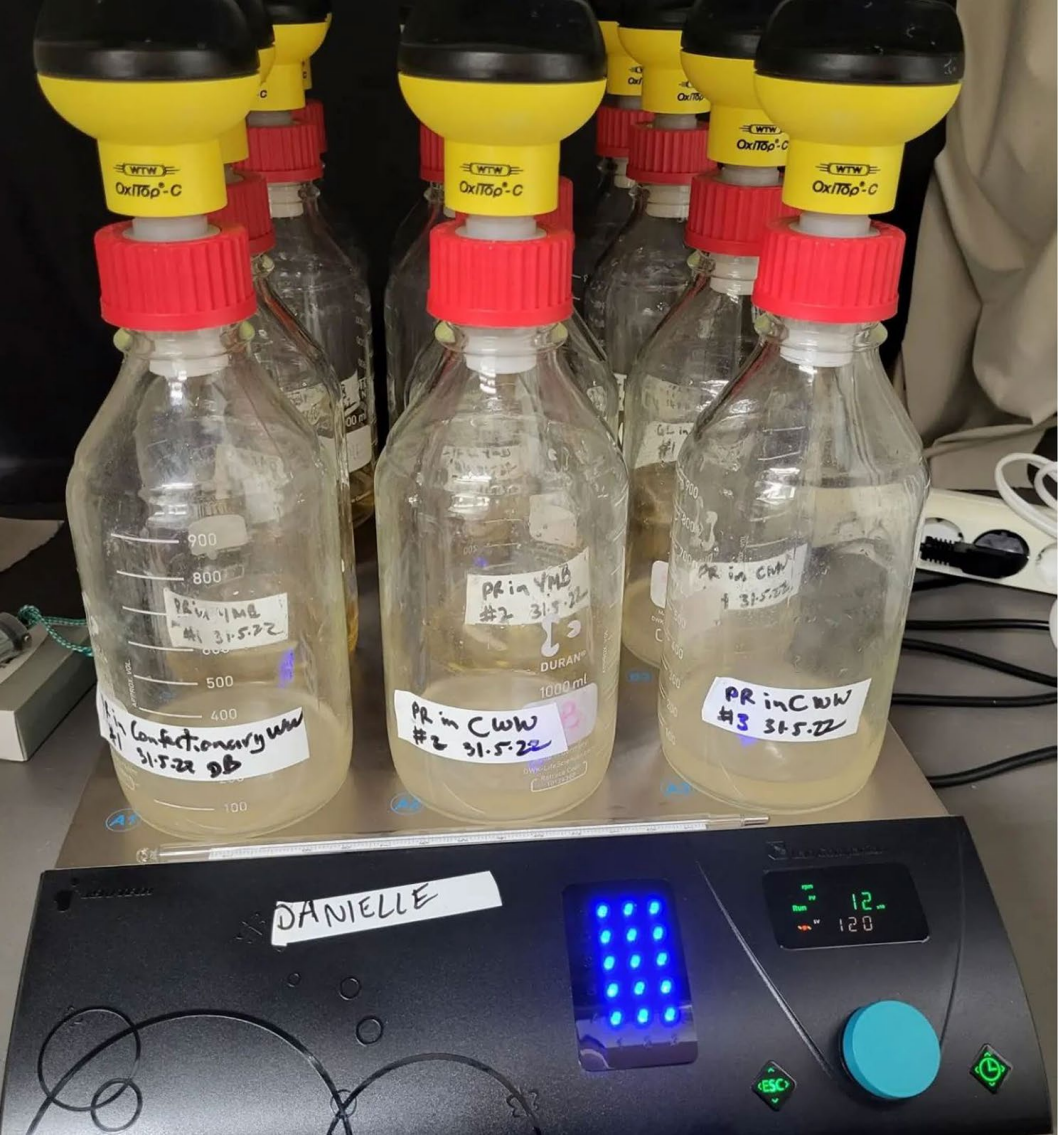
Oxygen uptake measurements using the OxiTop system

Oxygen uptake was measured using a manometric respirometer (OxiTop Control 12 system, WTW, Xylem Analytics, Germany) (Fig. 1), as a non-invasive determinant of metabolic activity in the cells [23, 24]. Each species was grown in batch cultures for 1–2 weeks and their oxygen uptake was continuously (56 min intervals) measured. Experiments were set up using aseptic techniques as follows: 0.5 mL (0.5 g DW (Dry Weight) L^− 1^) of culture was added to 80 mL of the WS in 1 L bottles. An antibiotic mix was added (P4083 Sigma-Aldrich) to suppress bacterial growth and ensure oxygen uptake was due only to the strains under investigation. Positive controls were set up similarly in 80 mL of YMB. Negative controls without fungi or yeast were set up for each WS. All cultures were carried out in replicates of four and incubated in the dark on a magnetic stirring platform at 120 rpm at room temperature (23–28 °C).

### Effect of pellet formation on oxygen uptake rates

Upon agitation some filamentous fungal species form pellets. This reduces oxygen availability to the cells within the pellets limiting their metabolism and growth [25]. To test the effect of pellet formation on oxygen uptake, agar (final concentration 2% v/v) was added to the WS and YMB (CWS/agar, Whey/agar, and YMB/agar) to inhibit pellet formation and allow for comparison of oxygen uptake between pelleted and non-pelleted samples. The three most pellet-forming species, *P. corylophilum*, *P. ostreatus* and *P. restrictum*, were tested. In the case of Whey and YMB, 2 g of agar powder (Fluka analytical) was added to 1 L of each WS and autoclaved as described above. Agar did not thicken under the highly acidic conditions of CWS (pH 3.5) so an 8% solution of agar in MQ was prepared and autoclaved. Then 250 mL of warm agar solution was combined with 750 mL of autoclaved CWS. The fungal species were then added to 80 mL of each WS with agar and set up in the manometric respirometer as described above. The pH of a fungal culture is an important parameter affecting growth and metabolic activity, so the pH of the substrates was measured before the addition of the fungi/yeast and at the end of the experiments.

### Statistical analysis

The mean maximum oxygen uptake rate (OUR) of each species/substrate combination was determined using linear regression and OURs were then divided into groupings of low (< 0.35 mg O_2_ L^− 1^ min^− 1^), moderate (0.35–0.55 mg O_2_ L^− 1^ min^− 1^) and high (> 0.55 mg O_2_ L^− 1^ min^− 1^). Differences between the OURs of a species in each substrate were tested using one-way ANOVA with Tukey’s post hoc tests (homogeneous variance), Welch’s one-way ANOVA with Games-Howell post hoc tests (homogeneity of variances violated) or Student’s t-test. The differences between OURs of cultures in pelleted vs. filamentous form were tested with Student’s *t*-test. Normality of distribution was tested with Shapiro–Wilk’s test and homogeneity of variances with Levene’s test in the case of both one-way ANOVA and Student’s *t*-test. Statistical procedures were conducted using SPSS version 28.

## Results

### Fungal growth trials

Biomass growth was the most rapid, abundant and sustained in Whey. When cultured in Whey seven species (*G. candidum*, *G. fermentans*, *G. lucidum*, *L. tigrinus*, *P. ostreatus*, *P. variotii*, *T. versicolor*) had increased biomass (more filamentous material, new growth spots, or formation of a mat on the surface) after only two days (Table 1). Six species (*G. fermentans*, *G. lucidum*, *L. tigrinus*, *P. ostreatus*, *P. variotii*, *T. versicolor*) continued to have increased biomass growth each week, becoming more viscous or having more growth spots/larger fungal mat for up to two weeks.

**Table 1:** Observations of fungal and yeast growth trials in five food production waste streams

	Time points (days) and waste streams														
	^1^T2					T7					T14				
Species	^2^Whey	CWS	BSW	PSW	RAS	Whey	CWS	BSW	PSW	RAS	Whey	CWS	BSW	PSW	RAS
Negative control															
*G. candidum*	^3^+	+		+	+		+	+	+	+	+		+	+	
*G. fermentans*	+			+		+	+	+			+	+	+		
*G. lucidum*	+			+		+	+	+	+		+		+		
*L. starkeyi*							+	+			+	+	+		
*L. tigrinus*	+		+	+		+	+	+	+		+	+		+	+
*P. corylophilum*			+	+		+	+	+			+	+			
*P. ostreatus*	+		+			+	+	+	+		+	+		+	+
*P. restrictum*			+	+			+	+			+				+
*P. variotii*	+		+			+	+	+	+		+				+
*T. versicolor*	+		+	+	+	+	+	+		+	+		+	+	

^1^ Observation time points: 2 days (T2), 7 days (T7) and 14 days (T14)

^2^ Cheese whey (Whey), confectionary/bakery waste stream, (CWS), barley steeping water (BSW), broad bean steeping water (PSW), recirculating aquaculture sludge (RAS)

^3^ Observable increase in fungal biomass (+)

Growth in CWS was slower with only one species, *G. candidum*, showing increased biomass after two days (Table 1). Only after a week did all species show biomass increases and half (*G. fermentans*, *L. starkeyi*, *L. tigrinus*, *P. corylophilum*, *P. ostreatus*) had substantially increased biomass at the end of two weeks which was very abundant (very viscous, or large fungal mats or numerous fungal spots covering almost the entire surface) and sustained.

In BSW six species (*L. tigrinus*, *P. corylophilum*, *P. ostreatus*, *P. restrictum*, *P. variotii*, *T. versicolor*) had increased biomass within two days only one of which, *T. versicolor*, continued to increase in biomass for two weeks (Table 1). However, at the end of one week, four other species (*G. candidum, G. fermentans, G. lucidum, and L. starkeyi*) had begun to show an increase in biomass with further biomass increases a week later. Overall, the biomass formed in BSW was not abundant i.e., cultures were not viscous, and the number of spots or size of the fungal mat was small.

Growth in PSW was rapid, seven species (*G. candidum, G. fermentans, G. lucidum, L. tigrinus, P. corylophilum, P. restrictum, and T. versicolor*) had increased biomass within two days (Table 1). However, biomass growth was neither sustained nor abundant and only two species, *G. candidum* and *L. tigrinus*, continued to have increases in biomass each week for up to two weeks. However, biomass growth was not abundant (not viscous, or the number of growth spots/size of the fungal mat was small) for any of the species which grew in PSW.

In RAS biomass growth was slow, neither abundant nor sustained. Only two species, *G candidum and T. versicolor*, had increased biomass in RAS after two days, however, biomass growth stopped after a week (Table 1). Only at the end of two weeks did four other species (*L. tigrinus, P. ostreatus, P. restrictum, and P. variotii*) begin to show increases in biomass. None of the species which grew in RAS were viscous or had a large fungal mat or numerous new growth spots.

Based on these results Whey and CWS were deemed most suitable for growth and chosen for further study and nutrient analysis. Whey was found to have remarkably high levels of COD, Total organic carbon (TOC), Total nitrogen (N-tot) and Total suspended solids (TSS) while phosphorus in the form of PO_4_-P was low. CWS had lower nutrient values than Whey (Table 2).

**Table 2:** Nutrient characteristics of cheese whey (Whey) and the confectionary/bakery waste stream (CWS)

Waste stream	COD (mg O_2_ L^-1^)	TOC (mg L^-1^)	N-tot (mg L^− 1^)	PO_4_-P (mg L^− 1^)	TSS (mg L^− 1^)
Whey	93 000	15 000	1 500	230	17 000
CWS	5 000	660	37	4	710

### Oxygen uptake rates

**Fig. 2:**
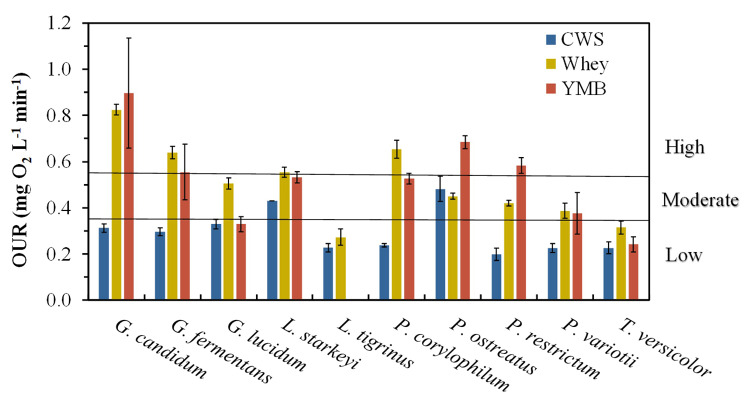
Mean maximum oxygen uptake rates (OURs) of all fungi and yeast species in three different substrates (mean ± SE, n = 4): cheese whey (Whey), confectionary/bakery waste stream (CWS), and culture broth – Yeast Malt Broth (YMB). *There was no growth of *L. tigrinus* in YMB

The OURs ranged from approx. 0.1 to 0.9 mg O_2_ L^− 1^ min^− 1^ and were generally higher in Whey and YMB compared to CWS (Fig. 2). The OURs were lowest in CWS where eight species had low OURs, and only two species (*L. starkeyi* and *P. ostreatus*) had moderate OURs. In Whey, only two species had low OURs (*T. versicolor* and *L. tigrinus*), four had moderate OURs and four had high OURs. In YMB one species (*L. tigrinus*) did not grow, two had low OURs, three had moderate OURs and four had high OURs (Fig. 2).

Seven species exhibited their highest OURs in Whey (Fig. 2). However, this was only significant for four species (*G. fermentans*, *p* < 0.001; *G. lucidum*, p = 0.002; *L. starkeyi*, p = 0.003; *P. corylophilum*, p < 0.001). Only for two species (*P. ostreatus, p* = 0.006 and *P. restrictum, p* < 0.001) were OURs significantly highest in YMB. *G. candidum, G. fermentans*, *G. lucidum*, *L. starkeyi, P. corylophilum*, and *P. restrictum* had significantly higher OURs in Whey than in CWS (*p*: <0.001, < 0.001, 0.003, 0.003, < 0.001 and < 0.001 respectively), while *G. lucidum* and *P. corylophilum* both had significantly higher OURs in Whey than in both CWS and YMB (*p*: 0.003, 0.003 & <0.001, 0.020 respectively). *P. ostreatus* and *P. restrictum* were the only species which had significantly higher metabolic rates in YMB than in both Whey (*p*: 0.007 and 0.004 respectively) and CWS (*p*: 0.015 and < 0.001 respectively), and none of the species had higher OURs in CWS than in Whey or YMB. *P. variotii* and *T. versicolor* had similar OURs in all WS (*p*: 0.115 and 0.123 respectively) and *G. candidum*, *G. fermentans* and *L. starkeyi* had similar OURs in Whey as in YMB (*p*: 0.952, 0.787 and 0.698 respectively). An additional table file gives more detail on all the statistics quoted above (see Additional file 1). Notably, of all the species, *G. candidum* had the highest metabolic rate in Whey and *L. tigrinus* did not grow at all in YMB.

### Effect of pelletization

There was little (< 10 pellets of < than 0.3 cm ø) or no pellet formation when filamentous fungi were grown in Whey. Conversely, in CWS more numerous and larger-sized (≥ 0.5 cm ø) pellets were formed. *P. corylophilum*, *P. ostreatus* and *P. restrictum* formed the largest and most numerous pellets in CWS and were chosen for comparison tests in Whey, CWS and YMB with agar.

**Fig. 3:**
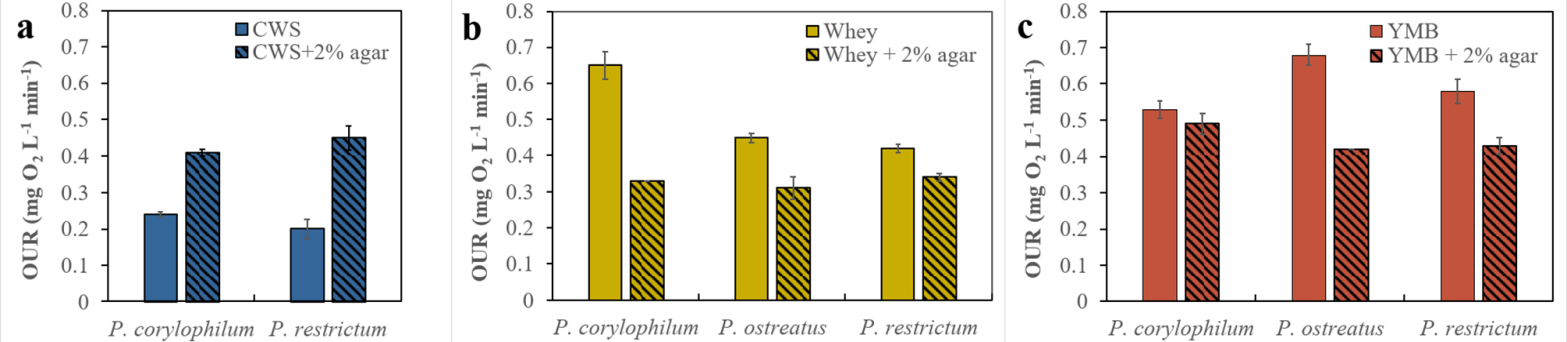
Mean maximum oxygen uptake rates (OURs) of *Penicillium corylophilum*, *Pleurotus ostreatus* and *Penicillium restrictum* in: (**a**) confectionary/bakery waste stream (CWS), (**b**) cheese whey (Whey) and (**c**)Yeast Malt Broth (YMB) with and without 2% agar (mean ± SE, n = 4). *Pleurotus ostreatus experiment in CWS was unsuccessful

*P. corylophilum* and *P. restrictum* in CWS/agar did not form pellets and their OURs were significantly greater (Fig. 3a) than when cultured w/o agar (*p*: < 0.001 and 0.004 respectively).

Conversely, *P. ostreatus* formed pellets in CWS/agar because the agar lost its viscosity. In Whey/agar and YMB/agar, the OURs of filamentous *P. corylophilum*, *P. ostreatus* and *P. restrictum* with agar were lower than w/o agar (Fig. 3b-c). The difference was significant for all species in Whey/agar vs. Whey (*P. corylophilum*: *p* = 0.014; *P. ostreatus*: *p* = 0.004; *P. restrictum*: *p =* 0.005) and for *P. ostreatus* (*p* = 0.012) and *P. restrictum* (*p* = 0.028) in YMB/agar vs. YMB. An additional table file gives more detail on all the statistics quoted above (see Additional file 2).

The biomass growth of *P. restrictum*, and *P. corylophilum* in YMB/agar was extremely abundant i.e., the entire culture volume was full of filaments and was visibly more abundant than in YMB w/o agar. An additional picture file illustrates the extremely abundant growth of *P. restrictum* in YMB/agar (see Additional file 3). They also formed more dense filamentous growth than in Whey/agar and CWS/agar, as illustrated in an additional picture file (see Additional file 4).

At the end of the experiments in CWS pH had increased from the initial pH of 3.5 by a change of > + 1 for most species with *L. starkeyi* and *P. restrictum* having the most marked increases in pH (as much as + 3 pH points) The pH of *G. lucidum* and *L. tigrinus* cultures had little or no change ( ≤ ± 0.5) and the pH of *P. ostreatus* cultures had decreased (a change of > -1) (Table 3).

**Table 3:** Modal pH of each species in each substrate at the end of experimental runs

Species	^1^CWS	Whey	YMB
*G. candidum*	5.5	6.0	5.5
*G. fermentans*	5.5	6.0	6.0
*G. lucidum*	4.0	4.5	3.5
* L. starkeyi*	6.5	6.0	5.0
* L. tigrinus*	3.5	5.5	5.0 *no biomass
*P. corylophilum*	6.0	8.0	4.5
*P. ostreatus*	2.0	5.0	2.0
*P. restrictum*	6.5	7.5	5.0
*P. variotii*	5.0	7.0	4.5
*T. versicolor*	5.5	5.5	5.0
	^2^CWS + 2% agar	Whey + 2% agar	YMB + 2% agar
*P. corylophilum*	4.0	7.5	4.5
*P. ostreatus*	2	5.0	3
*P. restrictum*	7	6.5	4.5

^1^ Confectionary/bakery waste stream (CWS), cheese whey (Whey), Yeast Malt Broth (YMB)

^2^ Confectionary/bakery waste stream with 2% agar (CWS + 2% agar), cheese whey with 2% agar (Whey + 2% agar), Yeast Malt Broth with 2% agar (YMB + 2% agar)

In Whey the pH of two species (*G. Lucidum and P. ostreatus*) decreased markedly from the initial pH of 6.5 (a change of -1.5–2), six species remained close to the initial pH (± 1) (*G. candidum, G. fermentans, L. starkeyi, L. tigrinus, P. restrictum, P. variotii, T. versicolor*) while only one species, *P. corylophilum*, increased pH by > + 1 pH point. Species grown in YMB generally remained close to the initial pH of 5–5.5 (± 1) except for two species where the pH decreased markedly (*G. lucidum* by ~ -2 points and *P. ostreatus* by ~ -3.5 points). When the three most pellet-forming species were grown in Whey/agar pH remained close (± 1) to its initial value of pH 6.5 except for *P. ostreatus* which decreased in pH by -1.5. On the contrary in CWS/agar pH increased (a marked change of + 3.5) from its initial value of pH 3.5 with *P. restrictum* cultivation but decreased (a change > -1) with *P. ostreatus* cultivation. In YMB/agar the only major pH change occurred with *P. ostreatus* cultivation where pH decreased notably from the initial value of pH 5–5.5 by ~ -2.5 points.

## Discussion

### Metabolic activity in different substrates

Initial growth screens were a fair indicator of each waste stream’s general suitability for fungal growth. Observations suggested that Whey and CWS were more appropriate fungal media than BSW, PSW and RAS. Based on these observations together with their high total organic carbon, Whey and CWS were chosen for further study. The OUR results suggested that Whey is generally a good growth medium for most fungi and yeasts. This is supported by five species having as good OURs in Whey as in the culture broth (positive controls) and two which had significantly higher OURs in Whey than in the culture broth. Additionally, Whey seems to be better for growth than CWS, as six species had higher metabolic activity in Whey than in CWS while none of the species had higher metabolic activity in CWS than in Whey. Also, none of the species had higher metabolic rates in CWS than the positive controls in the culture broth. Furthermore, most species had low metabolic rates (< 35 mg O_2_ L^− 1^ min^− 1^) in CWS while only two species had low metabolic rates in Whey suggesting that CWS performs more poorly as a growth medium overall.

The higher metabolic rates in Whey as opposed to CWS could be due to differing nutritional content. Bakery wastewater has high amounts of carbohydrates, natural oils, and fats [4], while confectionary wastewater consists mainly of sugars, fats, and dyes [26]. Whey is highly nutritious and is rich in sugars, proteins, vitamins, minerals, and growth factors such as insulin-like growth factor, platelet-derived growth factor, fibroblast growth factor, transforming growth factor and betacellulin [27, 28]. The COD values of the confectionary/bakery waste stream used here are comparable to previous values for bakery wastewater, 3000–7000 mg O_2_ L^− 1^ [4], but lower than those previously reported for confectionary wastewater, up to 20 000 mg O_2_ L^− 1^ [26]. However, its TOC content was much lower than its COD content suggesting low concentrations of bioavailable carbon. Its total nitrogen and phosphate concentrations were also low. In addition, CWS likely lacks vitamins and growth factors. Whey had much higher organic carbon, nitrogen, and phosphate concentrations than CWS (~ 23, 41 and 58 times higher respectively). It is possible that initial concentrations of carbohydrates, sugars, nitrogen, and phosphorus in CWS are high but were diluted during the washing of equipment [26], resulting in concentrations too low to support abundant growth. Also, the simple milk sugars in Whey will be more easily and quickly metabolized for respiration and biomass growth than more complex carbohydrates used in baked goods.

For some species, Whey may even be a better medium than the culture broth as *G. lucidum* and *P. corylophilum* had higher OURs in Whey than in YMB. Microbial media formulations are designed to contain the main macronutrients for the growth of a broad range of species. As such, they may lack the specific micronutrients and growth factors required by some species [29]. On the other hand, whey is a highly nutritious food substance containing many growth factors and may be more favourable than culture broth for some species. Other factors also affect growth and metabolism such as mixing speed, carbon source, pH, and pellet formation [30, 31]. Different carbon sources are assimilated by fungi and yeast to varying degrees and lactose, the carbon source in Whey, is not assimilated equally by all species [6, 32, 33]. For instance, *L. starkeyi* does not assimilate lactose at all and *P. ostreatus* does not assimilate lactose as well as other carbon sources [32, 34]. However, in this study, they had moderate metabolic rates in Whey, so if another more suitable carbon source were to be added to the Whey, growth and metabolic activity would have been even higher.

High mixing speed affects both pellet size (which affects oxygen availability) and dissolved oxygen concentration [35, 36], and plays a key role in the growth and metabolism of fungi. Hindered pellet formation of filamentous fungi in Whey is likely a key reason for the generally higher OURs in Whey as compared to CWS. Filamentous growth allows all the fungal biomass to access dissolved oxygen in the media while only the outer hyphae layer of pellets can access the dissolved oxygen [25]. However, the low pH of CWS may be a major factor affecting metabolic activity and probably growth in CWS [31]. Based on these factors and the fact that some species had similar metabolic rates in CWS and Whey, namely *P. ostreatus*, *L. tigrinus*, *P. variotii* and *T. versicolor*, CWS’s potential as growth media cannot be disregarded. Possible reasons for the lack of growth of *L. tigrinus* in YMB may be, (1) the formulation of YMB as it grows more slowly than the other species when maintained in YMB, and (2) too high mixing speed as it grew at a lower speed of 90 rpm in our previous study [19].

### Effect of growth mode and pH on metabolic activity

Generally higher OURs in Whey, where pelletization was suppressed, suggests that filamentous growth results in higher metabolic rates because of better access to dissolved oxygen unlike when grown in CWS or YMB where pellet formation occurred. The Whey was viscous and increased viscosity can result in disperse mycelial growth [24]. Whey had high TSS values, higher than previously reported, 8 000–11 000 mg/L [37]. Whey is also known to contain large amounts of proteins [38]. The suspended solids and coagulated proteins (due to autoclaving), when stirred, were distributed throughout the sample making it viscous and hindering pelletization [39]. Visual observations showed that CWS and YMB contained few particulates and had a consistency closer to water allowing for pellet formation. An additional picture file shows these viscosity and particulate observations (see Additional file 5). High ionic content can increase viscosity [40], and cheese whey contains many inorganic mineral ions, such as calcium, chloride, sodium, potassium, and magnesium [41], and organic and amino acids with charged functional groups [33, 42]. Yet another possible reason for pellet inhibition in Whey may be the carbon source as the type of carbon source affects fungal morphology [30]. Cheese whey contains 60–80% lactose [33], which induces filamentous growth in some species of fungi [30]. The combination of all these factors may induce filamentous growth in Whey leading to better oxygen availability and therefore higher metabolic activity.

Filamentous fungal growth in two of the three fungi tested in CWS/agar, *P. corylophilum* and *P. restrictum*, resulted in significantly higher OURs than pelleted growth in CWS w/o agar, further supporting the idea that filamentous growth results in higher metabolic activity. However, the addition of agar to Whey and YMB led to lower metabolic rates in *P. corylophilum*, *P. ostreatus* and *P. restrictum*. Oxygen availability is often the limiting factor in the growth and metabolism of submerged cultures [24]. Therefore, we expected filamentous growth, induced by the addition of agar, would lead to higher oxygen uptake rates than pelleted growth, where oxygen diffusion across the diffusive boundary layer may be rate limiting. However, pellet formation is not the only factor affecting oxygen availability. Oxygen availability is highly dependent on the concentration of dissolved oxygen governed by oxygen’s volumetric mass transfer rate from gas to liquid phase and the consumption rate of dissolved oxygen by the organism [24]. Volumetric mass transfer can be affected by culture conditions such as volume, aeration, mixing speed, and the physiochemical properties of the broth such as ionic strength, viscosity, and surface tension. All cultures in this study were enclosed batch cultures of the same volume stirred at the same speed. Therefore, any potential differences in the volumetric mass transfer of oxygen in the different substrates should be due to differences in the substrates’ physiochemical properties.

Based on visual observations substrate viscosity was in the order of CWS < YMB < Whey. This hierarchy is illustrated in an additional video file (see Additional file 6). The addition of agar resulted in the same order of viscosity, CWS/agar < YMB/agar < Whey/agar, which is also illustrated in an additional video file (see Additional file 7). Increasing viscosity decreases oxygen’s volumetric mass transfer rate [24, 39, 40], lowering oxygen availability. Increasing ionic strength has the same effect as it increases density and surface tension which lower mass transfer [40]. It is likely that the additive effects of agar and high ionic content, on density and surface tension, drastically reduced dissolved oxygen in Whey/agar. CWS however, with its lower viscosity and density, resulted in a final viscosity and density high enough to encourage filamentous growth, but not so high as to drastically hamper oxygen’s mass transfer. The presence of surfactants in CWS may also account for the higher OURs in CWS/agar.

Extremely dense filamentous growth in YMB/agar could explain the decreased OURs compared to pelleted growth in YMB w/o agar. Increased cell density increases viscosity causing uneven mixing and dissolved oxygen concentrations [30], pushing cultures into the stationary phase [31]. Since organisms’ oxygen uptake rate affects oxygen availability, extremely dense cultures can also lower dissolved oxygen concentrations. Altogether these factors could drastically decrease oxygen availability explaining the lower OURs in YMB/agar. Conversely, the same species in CWS/agar did not grow as densely so viscosity was not increased, and oxygen uptake was not high enough to drastically reduce dissolved oxygen concentrations. These results suggest there is a trade-off between having a sufficiently viscous medium to induce filamentous growth but not too viscous to hamper oxygen mass transfer.

The overall low metabolic rates obtained in CWS compared to Whey or YMB (with mostly moderate to high OURs) could be due to the extremely low pH of CWS. CWS contains detergents, some of which have low pH for disinfection purposes, explaining the low pH of CWS. Acidic pH favours pellet formation [36], which may be another reason, besides low viscosity, and surfactant content, for greater pellet formation in CWS resulting in low metabolic rates. Most fungi and yeast are acidophilic with an optimal pH range of 4–6. Many can grow under more acidic or alkaline conditions, but an intracellular pH of 5–6 is necessary for metabolism [31], making Whey and YMB better for metabolism and growth than CWS. Lower pH induces entry into the stationary phase [31], explaining the generally low metabolic rates obtained in CWS. Despite low pH, *L. starkeyi* and *P. ostreatus* did have moderate metabolic activity in CWS (43–48 mg O_2_ L^− 1^ min^− 1^). *L. starkeyi* is acidophilic (optimal pH range of 2.5–4) [43], making CWS suitable for *L. starkeyi’s* metabolism and growth. The pH range of *P. ostreatus* is 4–8 [34], but it had moderate to high metabolic activity despite lowering its culture pH in CWS, CWS/agar, YMB and YMB/agar, well below range, suggesting it tolerates extreme acidic conditions. Conversely, the pH of *P. ostreatus* in Whey and Whey/agar did not decrease below optimal range suggesting Whey has better buffering capacity than CWS. Yet another mark in favour of Whey as a more suitable growth medium. The fact that most of the cultures in CWS (except for *G. lucidum, P. ostreatus* and *L. tigrinus*) increased pH suggests that CWS’s pH was suboptimal, forcing them to employ energy-intensive strategies such as proton pumping or excretion of basic compounds, to increase pH at the cost of growth [31].

Growth of *P. ostreatus* in CWS/agar resulted in a loss of viscosity due to acidification by the fungus as agar cannot thicken under extremely acidic conditions [44]. Unlike *P. ostreatus*, *P. corylophilum* and *P. restrictum* increased pH which helped maintain viscosity in CWS/agar allowing for filamentous growth. The metabolic rates of *P. corylophilum* and *P. restrictum* were higher in CWS/agar than in CWS which shows that greater oxygen availability has a greater effect on metabolic activity than pH. The opposite was observed when agar was added to Whey and YMB, as all three species had lower metabolic rates than in Whey and YMB without agar. This surprising result is also due to oxygen availability, however, in this case, the increase in viscosity was too high reducing oxygen mass transfer and availability. These results are in line with previous research which shows oxygen availability to be the most crucial factor in the growth and metabolism of submerged fungi and yeast cultures [24]. Other factors such as temperature, age of inoculum, carbon dioxide levels and mixing speed can affect the metabolism of fungi and yeast [24, 31], any one of which could also be at play here. Temperature should not be an issue as the optimal temperature range for most yeasts and fungi is 22–30 °C [31, 45], and the temperature used here was within that range.

### Practical implications

The generally satisfactory metabolic rates in Whey, and to a lesser extent CWS, show their potential as growth substrates for fungi and yeast. Reuse of these FPWS via fungi/yeast biomass growth would produce value-added biomass and by-products increasing the circularity of water in food production. As the largest source of wastewater in food processing [46], the dairy industry in particular would benefit from a more circular production system. Whey contains high loads of BOD, COD, TSS, nitrogen, fats, oils and grease, the combination of which, presents challenges to treatment processes such as the clogging of wastewater treatment membranes [37]. The high fat content can also cause flotation of activated sludge particles which are then washed out of the treatment basin [46]. Treatment of whey via added-value fungal/yeast biomass growth could reduce these processing problems possibly producing high-value enzymes and biochemicals, reducing costs and increasing revenues. Though the quality, pH, and nutrient concentrations of CWS vary [26], it could also be reused if less sensitive acidophilic species were used, and culture parameters were optimized according to their requirements. The extremely acidic pH of CWS could be beneficial, preventing bacterial contamination [6], making this treatment system more feasible.

Though the manometric BOD measurement system used to gauge metabolic rates is readily available and easy to use, there were some limitations. For instance, accumulation of CO_2_ in the headspace and pelletization of filamentous fungal species lower metabolism [25, 47] so it is difficult to obtain a true measure of metabolic activity. As an indicator of growth, it is imperfect as cell activity slows when cultures become too dense. However, it does give a good overall indication of metabolic activity and possible growth in earlier non-oxygen-limited phases. Other limitations are specific to whey. One is its particulate nature, mainly due to protein aggregates which when autoclaved, would coagulate increasing viscosity. However, under real-world septic conditions, this will not be a problem. Another limitation specific to Whey is pH change. If whey sits for prolonged periods its pH drops due to fermentation [37], making it unsuitable for the growth of many fungi/yeasts, limiting it to onsite use.

Despite these limitations, the reuse of these two WS holds immense potential for nutrient removal via high-value fungal/yeast biomass growth while reducing treatment costs. The biomass could be used as fertilizer, biofuel feedstock or animal/fish feed provided there are no harmful contaminants. Depending on the species, industrially valuable enzymes and other biochemicals could be produced providing further economic gains to the system. For example, *P. corylophilum* produces cellulase, lipase, protease, xylanase, keratinase, pectinolytic enzymes and amylase which are used in the food (production of protein hydrolysates, baby formula, dietary supplements, processing of fruits, in baking) and fabric industries (laundry additive, leather and silk processing) [48]. Metabolic activity and growth of fungi/yeasts in these wastewaters could be further optimized by investigating the effect of mixing speed, pH, temperature, and pellet size on metabolic activity and by measuring the total biomass produced and its nutrient content. In addition to characterization of the biomass produced, characterization of all the waste streams before and after fungal growth would also give valuable information as to its bioremediation and nutrient recycling capabilities. This would allow for more definitive conclusions on the metabolic activity and potential for biomass growth and nutrient recovery.

## Conclusion

The findings of this study can be broken down into the following points:

- Out of the five FPWS, Whey and CWS were the most appropriate for fungal growth.
- Whey was generally a better growth media than CWS.
- Unexpectedly lower metabolic rates in Whey/agar and YMB/agar than in Whey and YMB without agar suggest that too high viscosity, and extremely abundant biomass growth in the case of YMB/agar, reduce metabolic processes despite filamentous growth.
- Pellet formation lowered metabolic activity and probably growth.

This study highlights the opportunity for water and nutrient circularity in food production, especially in the case of cheese production. Reuse of such FPWS is a step towards greater nutrient and water circularity in food production systems with potential cost savings and revenue from the biomass.

### Electronic supplementary material

Below is the link to the electronic supplementary material.

Supplementary Material 1

Supplementary Material 2

Supplementary Material 3

Supplementary Material 4

Supplementary Material 5

Supplementary Material 6

Supplementary Material 7

## Data Availability

The dataset(s) supporting the conclusions of this article is(are) included within the article (and its additional file(s)).

## References

[1] The United Nations world water development report 2020: Water and climate change. http://www.unwater.org/news/un-world-water-development-report-2020-%E2%80%98water-and-climate-change%E2%80%99. Accessed 10 July 2023.

[2] Hoekstra A (2013). The water footprint of modern consumer society.

[3] Mekonnen MM, Hoekstra AY (2011). The green, blue and grey water footprint of crops and derived crop products. Hydrol Earth Syst Sci.

[4] Asgharnejad H, Nazloo EKLMM, Hajinajaf N, Rashidi H (2021). Comprehensive review of water management and wastewater treatment in food processing industries in the framework of water-food-environment nexus. Compr Rev Food Sci Food Saf.

[5] Jin B, Yin P, Ma Y, Zhao L (2005). Production of lactic acid and fungal biomass by Rhizopus fungi from food processing waste streams. J Ind Microbiol Biotechnol.

[6] Sankaran S, Khanal SK, Jasti N, Jin B, Pometto ALI, Van Leeuwen HJ (2010). Use of filamentous fungi for wastewater treatment and production of high value fungal byproducts: a review. Crit Rev Environ Sci Technology.

[7] Sitharama A, Manatunge J, Ratnayake N, Nanayakkara CM, Jayaweera M (2017). Rapid degradation of FOG discharged from food industry wastewater by lipolytic fungi as a bioaugmentation application. Enviro Technol.

[8] Amann A, Zoboli O, Krampe J, Rechberger H, Zessner M, Egle L (2018). Environmental impacts of phosphorus recovery from municipal wastewater. Resour Conserv Recycl.

[9] Kaneko T, Kato H, Yamada H, Yamamoto M, Yoshida T, Attri P (2022). Functional nitrogen science based on plasma processing: quantum devices, photocatalysts and activation of plant defense and immune systems. Jpn J Appl Phys.

[10] Commodity. markets outlook. https://openknowledge.worldbank.org/server/api/core/bitstreams/813e7ba3-332f-55f0-a857-85c2cc773d7a/content. Accessed 24 May 2023.

[11] Zhongxiang Z, Yi Q (1991). Water saving and wastewater reuse and recycle in China. Water Sci Technol.

[12] Moreno-González M, Ottens M (2021). A structured approach to recover valuable compounds from agri-food side streams. Food Bioproc Tech.

[13] Hnain AK, Cockburn LM, Lefebvre DD (2011). Microbiological processes for waste conversion to bioenergy products: approaches and directions. Environ Rev.

[14] Li S, Zhao S, Yan S, Qiu Y, Song C, Li Y, Kitamura Y (2019). Food processing wastewater purification by microalgae cultivation associated with high value-added compounds production – a review. Chin J Chem Eng.

[15] Purchase D (2016). Fungal biology - fungal applications in sustainable environmental biotechnology.

[16] Rosa D, Duarte R, Saavedra IC, Varesche NK, Zaiat MB, Cammarota M (2009). Performance and molecular evaluation of an anaerobic system with suspended biomass for treating wastewater with high fat content after enzymatic hydrolysis. Bioresour Technol.

[17] Liu Z, Liu G, Cai H, Shi P, Chang W, Zhang S (2016). Paecilomyces Variotii: a fungus capable of removing ammonia nitrogen and inhibiting ammonia emission from manure. PLoS ONE.

[18] Moura MAFE, Martins BA, Oliveira GP, Takahashi JA (2022). Alternative protein sources of plant, algal, fungal and insect origins for dietary diversification in search of nutrition and health. Crit Rev Food Sci Nutr.

[19] Bansfield D, Spilling K, Mikola A, Piiparinen J (2022). Biofocculation of Euglena gracilis via direct application of fungal filaments: a rapid harvesting method. J Appl Phycol.

[20] Fasaei F, Bitter JH, Slegers PM, van Boxtel AJB (2018). Techno-economic evaluation of microalgae harvesting and dewatering systems. Algal Res.

[21] Leiva-Candia D, Pinzi S, Redel-Macías M, Koutinas A, Webb C, Dorado M (2014). The potential for agro-industrial waste utilization using oleaginous yeast for the production of biodiesel, fuel. Fuel.

[22] Risk Classification of Organisms: Pathogenicity classification of fungi. CGM/211004-01. https://cogem.net/app/uploads/2021/10/211004-01-Risk-Classification-of-Organisms-Fungi.-Status-October-2021.pdf. Accessed 5 February 2021.

[23] Sarrocco S, Gambineri SF, Magneschi L, Valentini G, Vannacci G (2008). Growth evaluation of an antagonistic Trichoderma virens isolate by using a BOD OxiTop respirometric apparatus. J Gen Appl Microbiol.

[24] Garcia-Ochoa F, Gomez E, Santos VE, Merchuk JC (2010). Oxygen uptake rate in microbial processes: an overview. Biochem Eng J.

[25] Pirt S (1966). A theory of the mode of growth of fungi in the form of pellets in submerged culture. Proc Royal Soc B.

[26] Zajda M, Aleksander-Kwaterczak U (2019). Wastewater treatment methods for effluents from the confectionery industry – an overview. J Ecol Eng.

[27] Bargeman G, Smith G (2003). Separation technologies to produce dairy ingredients. Dairy processing: improving quality.

[28] Michaelidou A, Steijns J (2006). Nutritional and technological aspects of minor bioactive components in milk and whey: growth factors, vitamins and nucleotides. Int Dairy J.

[29] Fries N (1948). The nutrition of fungi from the aspect of growth factor requirements. Trans Br Mycol soc.

[30] Espinosa-Ortiz EJ, Rene ER, Pakshirajan K, van Hullebusch ED, Lens PN (2016). Fungal pelleted reactors in wastewater treatment: applications and perspectives. Chem Eng J.

[31] Walker GM, White NA. Introduction to fungal physiology. In: Kavanagh K, editor. Fungi: Biology and applications. 3rd ed. John Wiley & Sons; 2018. pp. 1–34.

[32] Naganuma T, Uzuka Y, Tanaka K (1985). Physiological factors affecting total cell number and lipid content of the yeast, Lipomyces Starkeyi. J Gen Appl Microbiol.

[33] Vamvakaki A, Kandarakis I, Kaminarides S, Komaitis M, Papanikolaou S (2010). Cheese whey as a renewable substrate for microbial lipid and biomass production by Zygomycetes. Eng Life Sci.

[34] Adebayo-Tayo BC, Jonathan SG, Egbomuche OO, Egbomuche RC (2011). Optimization of growth conditions for mycelial yield and exopolysaccharride production by Pleurotus ostreatus cultivated in Nigeria. Afra j Microbiol res.

[35] Galaction A, Cascaval D, Oniscu C, Turnea M (2004). Prediction of oxygen mass transfer coefficients in stirred bioreactors for bacteria, yeasts and fungus broths. Biochem Eng J.

[36] Veiter L, Rajamanickam V, Herwig C (2018). The filamentous fungal pellet—relationship between morphology and productivity. Appl Microbiol Biotechnol.

[37] Shete BS, Shinkar N (2013). Dairy industry wastewater sources, characteristics and its effects on environment. Int j curr eng Technol.

[38] Jelen P, Tamime A (2009). Dried whey, whey proteins, lactose and lactose derivative products. Dairy powders and Concentrated products.

[39] Gibbs P, Seviour R, Schmid F (2000). Growth of filamentous fungi in submerged culture: problems and possible solutions. Crit Rev Biotechnol.

[40] Ferreira P, Lopes M, Mota M, Belo I (2016). Oxygen mass transfer impact on citric acid production by Yarrowia Lipolytica from crude glycerol. Biochem Eng J.

[41] Carvalho F, Prazeres AR, Rivas J. Cheese whey wastewater: characterization and treatment. Sci Total Environ. 2013;445–6:385 – 96. 10.1016/j.scitotenv.2012.12.038 23376111

[42] Gernigon G, Piot M, Beaucher E, Jeantet R, Schuck P (2009). Physicochemical characterization of mozzarella cheese wheys and stretchwaters in comparison with several other sweet wheys. J Dairy Sci.

[43] Koenig DW, Day DF (1988). Production of Dextranase by Lipomyces Starkeyi. Biotechnol Lett.

[44] Kanazawa S, Kunito T (1996). Preparation of pH 3.0 agar plate, enumeration of acid-tolerant, and Al-resistant microorganisms in acid soils. Soil Sci Plant Nutr.

[45] Ali SR, Fradi AJ, al-Aaraji AM (2017). Effect of some physical factors on growth of five fungal species. Euro Acad Res.

[46] Slavov AK (2017). General characteristics and treatment possibilities of dairy wastewater – A review. Food Technol Biotechnol.

[47] Waid J (1962). Influence of oxygen upon growth and respiratory behaviour of fungi from decomposing rye-grass roots. Trans Br Mycol soc.

[48] Yadav AN, Singh S, Mishra S, Gupta A (2019). Recent Advancement in White Biotechnology through Fungi: perspective for value-added products and environments.

