# Supplementary material for: Growth of fungi and yeasts in food production waste streams: a feasibility study
Source: BMC Microbiol. 2023 Nov 6;23:328. doi: 10.1186/s12866-023-03083-6 (PMC10626767; doi:10.1186/s12866-023-03083-6)
Supplement: Supplementary file 1 — Supplementary Material 1 [file 12866_2023_3083_MOESM1_ESM.pdf]

Additional file 1. Comparison of the difference in mean maximum oxygen uptake rates (OUR) (one-way ANOVA with Tukey's post hoc test, Welch's one-way with Games-Howell post hoc tests and student's t-test) of each species when cultured in cheese whey (Whey), confectionary/bakery waste stream (CWS) and culture broth (Yeast Malt Broth (YMB)) (mean ± SE, n = 4). + denotes an increase in the expected direction. - denotes a decrease in the expected direction.

	Pairwise comparison difference in means					
Species	Whey>CWS	YMB>Whey	YMB>CWS	df	F/t	*p*
G. candidum	+0.51 ± 0.03 (*p* <0.001)	+0.07 ± 0.24 (*p* = 0.952)	+0.59 ± 0.24 (*p* = 0.175)	2,5.25	126.31	<0.001
G. fermentans	+0.34 ± 0.03 (*p* < 0.001)	-0.09 ± 0.12 (*p* = 0.787)	+0.26 ± 0.12 (*p* = 0.237)	2,5.15	52.91	<0.001
G. lucidum	+0.18 ± 0.04 (p = 0.003)	-0.18 ± 0.04 (p = 0.003)	0.00 ± 0.04 (p = 1.000)	2,9	14.08	0.002
L. starkeyi	+0.13 ± 0.03 (*p* = 0.003)	-0.02 ± 0.03 (*p* = 0.698)	+0.10 ± 0.03 (*p* = 0.011)	2,9	11.95	0.003
L. tigrinus	+0.05 ± 0.04	NA	NA	6	1.13	0.301
P. corylophilum	+0.42 ± 0.03 (*p <0.001*)	-0.13 ± 0.03 (*p* = 0.020)	+0.29 ± 0.03 (*p* <0.001)	2,7	88.07	<0.001
P. ostreatus	-0.03 ± 0.05 (*p* = 0.801)	+0.23 ± 0.05 (*p* = 0.007)	+0.20 ± 0.05 (*p* = 0.015)	2,8	10.30	0.006
P. restrictum	+0.22 ± 0.03 (*p* <0.001)	+0.16 ± 0.03 (*p* = 0.004)	+0.38 ± 0.03 (*p* <0.001)	2,7	61.44	<0.001
P. variotii	+0.18 ± 0.9 (*p* = 0.169)	-0.01 ± 0.09 (*p* = 0.994)	+0.17 ± 0.08 (*p* = 0.157)	2,8	2.87	0.115
T. versicolor	+0.09 ± 0.04 (*p* = 0.128)	-0.07 ± 0.04 (*p* = 0.238)	+0.02 ± 0.04 (*p* = 0.907)	2,9	2.67	0.123
