# Supplementary material for: Growth of fungi and yeasts in food production waste streams: a feasibility study
Source: BMC Microbiol. 2023 Nov 6;23:328. doi: 10.1186/s12866-023-03083-6 (PMC10626767; doi:10.1186/s12866-023-03083-6)
Supplement: Supplementary file 2 — Supplementary Material 2 [file 12866_2023_3083_MOESM2_ESM.pdf]

Additional file 2. The differences in mean maximum oxygen uptake rates (Student's t-test) of *Penicillium corylophilum, Pleurotus ostreatus* and *Penicillium restrictum* when grown in confectionary/bakery waste stream (CWS), cheese whey (Whey) and culture broth i.e., Yeast Malt Broth (YMB) - with and without agar added (mean ± SE, n = 4). + denotes an increase in the expected direction. - denotes a decrease in the expected direction.

Species	Pairwise comparison	Difference in means	df	t	*p*
P. corylophilum	CWS/agar > CWS	+0.17 ± 0.01	5	14.14	< 0.001
	Whey/agar >Whey	-0.32 ± 0.04	2	-8.41	0.014
	YMB/agar >YMB	-0.06 ± 0.04	5	-1.35	0.234
P. ostreatus	Whey/agar >Whey	-0.14 ± 0.03	5	-5.05	0.004
	YMB/agar >YMB	-0.26 ± 0.03	2	-8.89	0.012
P. restrictum	CWS/agar > CWS	+0.25 ± 0.04	4	5.95	0.004
	Whey/agar >Whey	-0.08 ± 0.02	5	-4.78	0.005
	YMB/agar >YMB	-0.15 ± 0.04	4	-3.36	0.028
