# Supplementary material for: Growth of fungi and yeasts in food production waste streams: a feasibility study
Source: BMC Microbiol. 2023 Nov 6;23:328. doi: 10.1186/s12866-023-03083-6 (PMC10626767; doi:10.1186/s12866-023-03083-6)
Supplement: Supplementary file 3 — Supplementary Material 3 [file 12866_2023_3083_MOESM3_ESM.pdf]

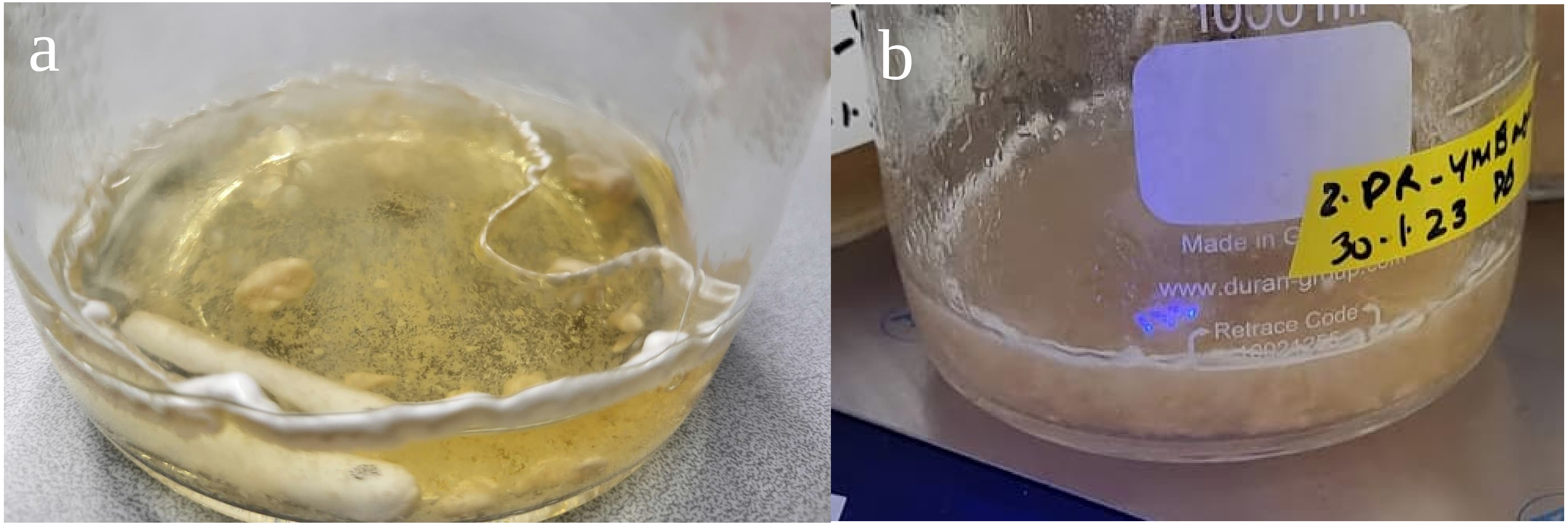

Additional file 3. *P. restrictum* cultured in Yeast Malt Broth (YMB) with and without agar: (a) *P. restrictum* in YMB, (b) *P. restrictum* in YMB + 2% agar.
