# Supplementary material for: Growth of fungi and yeasts in food production waste streams: a feasibility study
Source: BMC Microbiol. 2023 Nov 6;23:328. doi: 10.1186/s12866-023-03083-6 (PMC10626767; doi:10.1186/s12866-023-03083-6)
Supplement: Supplementary file 4 — Supplementary Material 4 [file 12866_2023_3083_MOESM4_ESM.pdf]

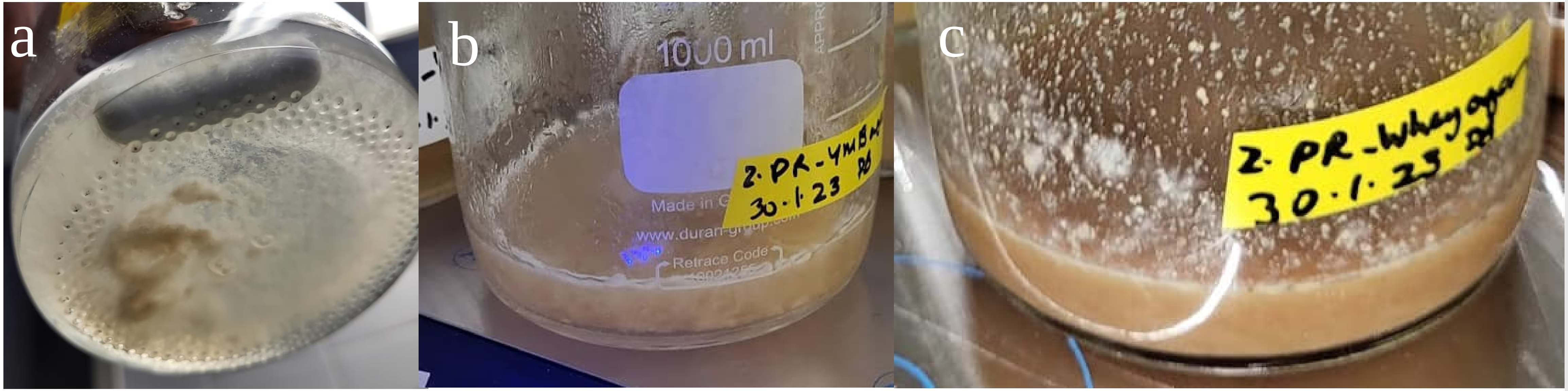

Additional file 4. *P. restrictum* cultured in three different substrates with agar: (a) confectionary/bakery waste stream (CWS) + 2% agar, (b) Yeast Malt Broth (YMB) + 2% agar, (c) cheese whey (Whey) + 2% agar
