# Supplementary material for: Growth of fungi and yeasts in food production waste streams: a feasibility study
Source: BMC Microbiol. 2023 Nov 6;23:328. doi: 10.1186/s12866-023-03083-6 (PMC10626767; doi:10.1186/s12866-023-03083-6)
Supplement: Supplementary file 5 — Supplementary Material 5 [file 12866_2023_3083_MOESM5_ESM.pdf]

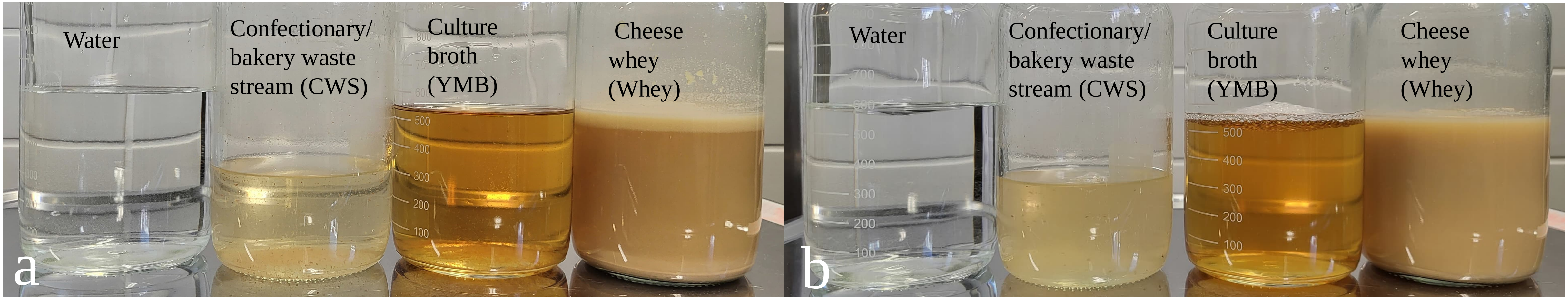

Additional file 5. Pictures of each substrate in comparison to water: a) before shaking, b) after shaking.
