# Supplementary material for: Growth of fungi and yeasts in food production waste streams: a feasibility study
Source: BMC Microbiol. 2023 Nov 6;23:328. doi: 10.1186/s12866-023-03083-6 (PMC10626767; doi:10.1186/s12866-023-03083-6)
Supplement: Supplementary file 7 — Supplementary Material 7 [file 12866_2023_3083_MOESM7_ESM.pptx]

## Slide 1
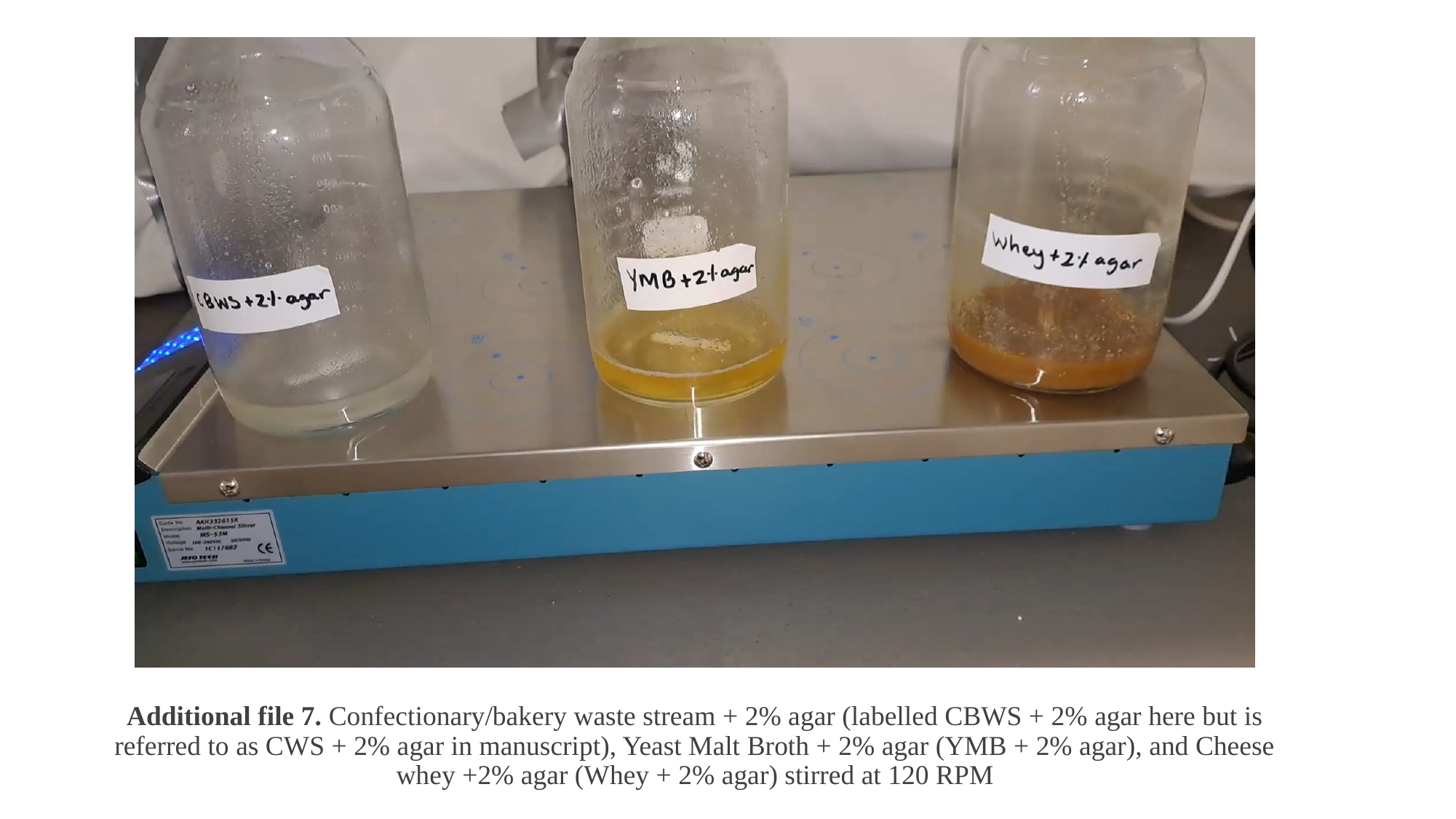

Additional file 7. Confectionary/bakery waste stream + 2% agar (labelled CBWS + 2% agar here but is referred to as CWS + 2% agar in manuscript), Yeast Malt Broth + 2% agar (YMB + 2% agar), and Cheese whey +2% agar (Whey + 2% agar) stirred at 120 RPM
